# Micro-Polluted Surface Water Treated by Yeast-Chitosan Bio-Microcapsules

**DOI:** 10.3390/ma13163519

**Published:** 2020-08-10

**Authors:** Xiao Liu, Lin Wang, Jun Shi

**Affiliations:** 1 Ren’ai College, Tianjin University, Tianjin 301636, China; tjliuxiao80@163.com; 2 Key Laboratory of Yangtze River Water Environment Ministry of Education, College of Environmental Science and Engineering, Tongji University, Shanghai 200092, China; wl87021@sina.com

**Keywords:** *Candida tropicalis*, suspended solids, ammonia nitrogen, nutrient substrate, nitrite

## Abstract

Ammonia nitrogen and natural organic matter (NOM) seriously degrade the quality of surface waters. In this study, the optimum preparation conditions of a yeast-chitosan bio-microcapsule of the *Candida tropicalis* strain, used to treat micro-polluted surface water, were investigated. Fourier transform infrared spectroscopy and scanning electron microscopy were used to characterize the bio-microcapsules. A continuous laboratory-scale reaction apparatus was built to evaluate the engineering applications of the bio-microcapsules and their treatment efficiency for major pollutants in micro-polluted raw water. The yeast-chitosan bio-microcapsules were found to rapidly and effectively remove suspended solids and ammonia nitrogen. Moreover, the bio-microcapsule pre-treatment process was capable of resisting impact loads and fluctuations in water quality. Even at low temperatures (12 °C), the removal rate of ammonia nitrogen still reached 79%. The treatment did not lead to a temporary increase in nitrite concentration, nor to the excessive accumulation of nitrogen. The application of bio-microcapsules is simple; it only requires aeration and certain nutrient substrates, and can be adapted to treat raw drinking water with a poor nutrient substrate, therefore showing promise for future use in engineering applications.

## 1. Introduction

Ammonia nitrogen and natural organic matter (NOM) are a great threat to the quality of surface water [1]_._ Micro-polluted surface water refers to surface water slightly contaminated by organic matter and ammonia [2]. Ammonia is an environmental toxicant that is particularly problematic for aquatic organisms [3], and its incomplete nitrification increases toxic nitrite contents, such as nitrate nitrogen and nitrite nitrogen [4]. Chlorine is the most commonly used disinfectant in water treatment [5]; however, free chlorine can react with NOM to form disinfection by-products (DBPs) [6]. Moreover, some NOM components can react with disinfectants to form disinfection by-products such as trihalomethanes (THMs), which are hazardous to health and have associated drinking water guideline values [4]. Another important problem related to NOM is microbial regrowth in water distribution systems, which has adverse effects on treated water quality [7]. Therefore, there is an urgent need to develop cost-effective processes for the removal of ammonia nitrogen and NOM from drinking water.

Flocculation-filtration-disinfection, as a conventional treatment process, does not effectively remove these contaminants. Micro-polluted surface water is commonly treated by adsorption [8], catalytic oxidation and membrane filtration. However, these approaches have shortcomings: adsorption accumulates the contaminants but cannot completely degrade the micropollutants; catalytic oxidation is limited by complex technology; and membrane filtration is restricted by expensive operation costs, as well as fouling problems [9]. Compared to these technologies, biodegradation is considered more efficient for the removal of organic carbon and ammonia nitrogen from micro-polluted surface water [10,11].

As biodegrading microorganisms, yeasts have the characteristics of strong environmental adaptability and acid resistance [12], and show high tolerance to a high concentration of organic substrates, strong metabolism and the rapid degradation of organic matter. Currently, there is a lot of research regarding the treatment of industrial, municipal and agricultural wastewaters using yeast species such as *Candida halophila* and *Rhodotorula glutinis*, *Pichia*, *Trichosporon*, and some unidentified *Ascomycetes* [13]. The binary yeast isolates *Candida halophila* and *Rhodotorula glutinis* achieved 85% chemical oxygen demand (COD) reduction from glutamate fermentation wastewater [14]. *Pichia guilliermondii* was able to remove approximately 90% biochemical oxygen demand (BOD) within 24 h in waste brine generated from kimchi production [15]. Both *Trichosporon oivide* and *Trichosporon cutaneum* are capable of degrading phenol and chlorophenol [13]. It was observed that the yeast *Candida bombicola* can completely remove oils and fats (>95% COD removal) under continuous and batch operation [16]. Tan et al. found that the yeast strain LH-F1 can decolorize various azo dyes (20 mg/L) through adsorption and degradation [17]. Moreover, *Candida tropicalis* cells, which are mainly used as biosorbents for heavy metal decontamination because of their resistance, phenol degradation and diesel oil degradation as biofilms, are capable of adsorbing Cu(II) and phenol in both single and binary systems [18]. However, the use of free yeast cells for pollutant removal is often sensitive to important biotic and abiotic factors in the environment (e.g., high/low temperature and pH conditions) [19]. Furthermore, collection and recycling issues limit the application of free yeast cells.

Compared to free cells, the use of immobilized or encapsulated cells is considered more effective, as it leads to higher biomass loading, easier operation, higher biodegradation rates, and greater protection from toxic substances [20]. To improve the adaptation of free cells and to enhance the bioremediation of organic pollutants, different encapsulation methods have been developed [21]. Sodium alginate-chitosan bio-microcapsules are some of the most commonly used bio-microcapsules, because the procedure is simple, has relatively mild effects, and is non-toxic to cells [22]. Chitosan is a naturally occurring carbohydrate polymer that displays the following characteristics: it is biocompatible, biodegradable, non-toxic to humans and the environment, and shows antimicrobial and antioxidant activities [23]. Bio-microcapsules comprise one of the preferred systems for cell culture [24], and represent an exciting biotechnology approach to fermentation [25], especially for the production and intestinal delivery of therapeutic agents from genetically modified food-grade microorganisms [26,27]. However, yeast bio-microencapsulation has not previously been utilized to remove ammonia nitrogen and NOM from micro-polluted surface waters.

In this study, a yeast-chitosan bio-microcapsule of the *Candida tropicalis* strain was prepared and a set of dynamic small-scale experimental setups were established to investigate the treatment effect of micro-polluted raw water. The objectives of this work were: (1) to determine the best preparation conditions through the orthogonal experiment, focusing on four factors, namely curing time, sodium alginate concentration, calcium chloride and chitosan concentrations, and the mechanical strength and permeability of microcapsules; (2) to characterize the bio-microcapsules using Fourier transform infrared (FTIR) spectroscopy and scanning electron microscopy (SEM); and (3) to assess the removal efficiency of turbidity, ammonia nitrogen, nitrite nitrogen, nitrate nitrogen and COD_Mn_.

## 2. Materials and Methods

### 2.1. Materials and Standards

YPD medium (Y1500, 1% yeast extract, 2% peptone and 2% glucose) was purchased from Sigma Aldrich, St. Louis, MI, USA.

The dry powder of *Candida tropicalis* strain CICC1351 was provided by the China Center of Industrial Culture Collection, Beijing, China. The dry strain powder was dissolved in a small amount of sterilized deionized water, then approximately 10 mL of maltose culture medium was added, before being placed it in a constant temperature air bath incubator and cultured at 25 °C for 3 days. Next, 1 mL of the activated strain solution was transferred to 150 mL of the newly prepared maltose medium, and then cultured in a constant temperature air bath at 25 °C and 200 rpm for 12 h. The growth of the strain reached the best activity, which could be used for the experiments.

Sodium alginate and chitosan are the main materials in microcapsules. Sodium alginate was purchased from Sigma Aldrich, USA. Chitosan was selected with a high deacetylation degree (>80%) and a low molecular weight (MV = 300,000), and was purchased from Qianguang Bioengineering Co., Ltd., Qingdao, China and dissolved in 1% acetic acid in a 60 °C constant temperature water bath. Glutaraldehyde (25%) was the cross-linking agent and calcium chloride was the fixed agent, both of which were purchased from Sinopharm Chemical Reagent Co., Ltd., Shanghai, China. 

Ammonia nitrogen standard stock solution was achieved by the following process: First, the concentration was 1 mg/mL, and the appropriate amount of high-grade pure ammonium chloride (NH_4_Cl) was weighed and dried at 100–105 °C for 2 h. Then 3.8190 g of NH_4_Cl was weighted and dissolved in a small amount of non-ammonia water, which was then transferred into a 1000 mL volumetric flask to ensure a constant volume that could be stored for one month at 2–5 °C. NH_4_Cl was purchased from Sinopharm Chemical Reagent Co., Ltd., China.

For the physiological saline (0.9%, m/v), 9 g NaCl was dissolved in a small amount of distilled water, and then transferred into a 1000 mL volumetric flask to ensure a constant volume. After sterilization at 121 °C for 15 min, it was cooled to ambient temperature and stored in a refrigerator. NaCl was purchased from Sinopharm Chemical Reagent Co., Ltd., China.

All of the above reagents were of analytical pure grade.

### 2.2. Preparation of the Bio-Microcapsules

#### 2.2.1. Yeast Extraction

First, 20 mL of bacteria culture fluid in the logarithmic growth phase was placed in a dry refrigerated centrifuge(Thermo KR25i, Thermo Fisher Scientific, Walsham, MA, USA), and then centrifuged for 5 min at 4000 rpm (1776× *g*) and 15 °C. Next, the supernatant was poured off and the remnant was washed three times with physiological saline. Finally, the strains were collected by suspended extraction using 10 mL of physiological saline, and then stirred with a vortex mixer(vortex-genie 2, Scientific Industries, Hamilton, NJ, USA). The OD600 of the solution was determined as approximately 1.5 [28,29].

#### 2.2.2. Preparation of the Bio-Microcapsules

Two steps were included in the preparation of the sodium alginate-chitosan microcapsules: (1) the sodium alginate solution was blended with the yeast strains at a certain percentage, then calcium chloride solution was added to the mixture in drops to form gel beads of calcium alginate; and (2) the gel beads were polymerized with chitosan to form microcapsules. If required, chemical modification with a cross-linking agent (e.g., TAIC) can be performed during the subsequent step.

In step 1, the sodium alginate solution was blended with the yeast strains at a certain percentage. A 20 mL syringe was used to extrude the mixed liquid into the CaCl_2_ solution at a uniform velocity. The titration height (i.e., the distance between the syringe and the liquid surface of the CaCl_2_ solution) was controlled at 5–6 cm to obtain spherical microcapsules with a uniform particle size. After a period of solidification, a 150 mesh filtration screen was applied to obtain bio-microcapsules, which were then rinsed three times with deionized water to remove residual CaCl_2_ from the capsule surface. The calcium alginate gel beads were placed in the chitosan solution until coated, then filtrated and washed three times to remove excess chitosan from the surface. Then, the alginate-chitosan bio-microcapsules were placed in a cross-linking agent (glutaraldehyde) solution to enhance their mechanical strength, followed by filtration and three instances of flushing, before being placed in physiological saline for backup. The full preparation process was conducted on a sterile operation platform (SW-CJ-1D, Jiangsu Tongjing Co., Ltd., Nantong, China).

### 2.3. Experimental Set-Up and Conditions

**Figure 1:**
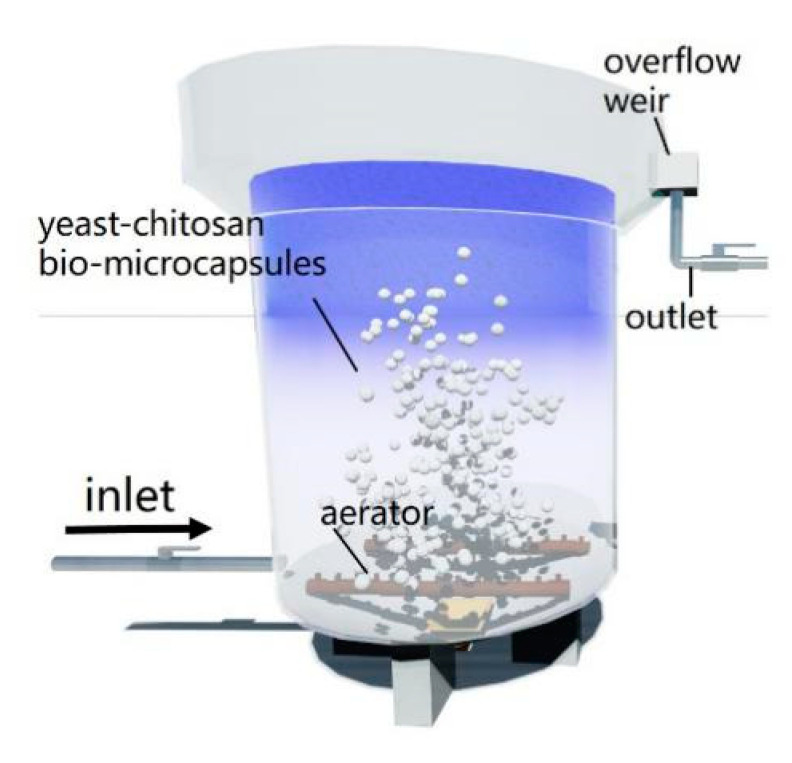
Bench-scale experimental setup: A cylindrical contact reaction tank connected with the membrane tank.

The raw water was taken from Sanhaowu River in the campus of Tongji University. The water quality of Sanhaowu River is shown in Table 1, a typical micro-polluted raw water. The raw water was stored in the feeding tank before being extruded to the bottom of a contact tank (containing pre-dosed bio-microcapsules) through a feed pump. Diffusers were set in the contact tank, the gas supply and aeration were engaged by an oxygenating pump, and the bio-microcapsules and raw water were thoroughly mixed during oxygenation (Figure 1).

### 2.4. Chemical Analysis

The water quality was analyzed by measuring the chemical parameters, including turbidity, ammonia nitrogen (NH_3_–N), nitrite nitrogen (NO_2_–N), nitrate nitrogen (NO_3_–N) and the permanganate index (COD_Mn_). In this study, COD_Mn_ indicated the concentration of organic compounds. The analytical protocol followed the standard methods of the State Environmental Protection Administration of China [30]. NH_3_-N was determined by Nessler’s reagent spectrophotometry, while NO_2_–N was determined by N-(1-naphthyl)-ethylenediamine spectrophotometry, and NO_3_–N by ultraviolet spectrophotometry using a spectrometer (GENESYS 10S, Thermo Fisher Scientific, Walsham, MA, USA). COD_Mn_ was quantified by the KMnO_4_ oxidation method, TOC was determined by TOC analyzer (Shimadzu, Kyoto, Japan), while a turbidimeter (2001P, HACH, Loveland, CO, USA) was used to monitor turbidity, and a pH meter (FE28, Mettler Toledo, Zurich, Switzerland) was used to record the pH value. All water samples were filtered through a 0.45 μm syringe filter (Whatman, Maidstone, UK) prior to analysis, in order to remove matter that could inhibit the accuracy of the analysis. Three samples were taken at every turn and the average value was calculated.

### 2.5. FTIR Spectroscopy

The IR spectra of the bio-microcapsules were recorded with an FTIR spectrometer (Nicolet 5700, Thermo Fisher Scientific, USA) over a frequency range of 4000–625 cm^−1^.

### 2.6. Scanning Electron Microscopy

The surface characteristics of the bio-microcapsules were imaged by a scanning electron microscope (SEM-515, Philips, Amsterdam, The Netherlands) operated at 15 kV. The bio-microcapsules were dispersed in 10 mL of NaCl solution (4 mM) and incubated while being shaken at 100 rpm (44× *g*) for 2 h at 25 °C. The dispersion was dropped on metal stubs and airdried at ambient temperature (25 °C). The samples were mounted to the specimen holder with a double-sided adhesive tape and vacuum-coated with gold for a period of 30 s before SEM imaging [22,31].

### 2.7. Measurement of the Mechanical Strength and Permeability

The mechanical strength of the bio-microcapsules was characterized by the maximum pressure on the front of the bio-microcapsules. First, *n* bio-microcapsules were randomly taken and placed onto the electronic balance tray (UW6200H, Shimadzu, Kyoto, Japan), evenly distributed into a circle. Then, the tray was covered and a slide was pressed on it, the balance reading was set to zero, the slide was pressed with fingers until the capsule had serious deformation or cracks, and the total mass Pi was read out. The mechanical strength P of a single bio-microcapsule was ∑Pi/*n*. At least three groups of data were measured, and the average value was the average mechanical strength of the bio-microcapsule.

In this experiment, the permeability of the bio-microcapsules was determined via the method of ink penetration. Capsules with uniform particle size were taken and placed into the ink, taking out *n* capsules every 5 min, which were frozen at 4 °C for 5 min. Next, the diameter d_1i_ was measured with a Vernier caliper, before being cut open to observe the vertical section, where the circular diameter d_2i_ in the center (not stained by the ink) was measured with a Vernier caliper. Then, the permeability W was equal to 100% (d_1i_ − d_2i_)/d_1i_.

The greater permeability of the bio-microcapsule indicates that the faster the penetration speed, the better the permeability, which is conducive to the material exchange inside and outside the membrane, and ensures the nutritional metabolism of microorganisms.

### 2.8. Data Analysis

The calculations were as follows: chemical remaining percentage (%) = (residue chemical concentration/initial chemical concentration) × 100%; chemical removal rate (%) = [1 − (residue chemical concentration/initial chemical concentration)] × 100%. Significant differences were accepted at *p* < 0.05.

## 3. Results and Discussion

### 3.1. Optimum Microcapsule Preparation Conditions

Since the sodium alginate microcapsules were formed by the gelation between sodium alginate and calcium ions [32], the concentrations of sodium alginate and CaCl_2_ are critical factors for yeast immobilization. The other factors tested included solidifying time and coating time. The optimum conditions for the preparation of the yeast-chitosan bio-microcapsules were obtained by means of an orthogonal experiment, a useful method for determining the optimum conditions and levels of importance of different factors in a process [22]. A L_16_ (4^4^) orthogonal design was applied to optimize these parameters. Mechanical strength was used as an optimization index for the orthogonal array analysis (Table 2). The level of the parameter resulting in the highest mechanical strength refers to the most favorable level of that parameter in the bio-microcapsule preparation process, and the information presented in Table 2 shows that the optimum conditions for bio-microcapsule preparation were: (1) Sodium alginate concentration = 2% (w/v); (2) calcium chloride concentration = 5% (w/v); (3) curing time = 30 min; and (4) film overlaying time = 10 min. The level of importance based on the orthogonal experiment was in the following order: sodium alginate concentration > calcium chloride concentration > curing time > film overlaying time. This means that the concentration of sodium alginate was the greatest influencing factor, playing a critical role in the overall properties of the bio-microcapsules, which is consistent with the results of Lu et al. [20]. With an increase in the concentration of sodium alginate, the capsule size increased significantly, and the shape became rounder and more uniform. This is because an increase in the concentration of sodium alginate leads to an increase of raw material density, and an exponential increase in surface tension, causing a volume increase in the droplets. Therefore, the particle size of the solidified microcapsules increased. Furthermore, an increase in surface tension also ensures the uniform roundness of the microcapsules. In the experiment, the sodium alginate solution was mixed with a certain percentage of yeast liquid, which decreased the concentration of sodium alginate, thereby having a negative effect on the preparation of the bio-microcapsules. Therefore, the bio-microcapsules were prepared using 3% sodium alginate in the following experiments.

### 3.2. Characterization of the Bio-Microcapsules

#### 3.2.1. SEM

**Figure 2:**
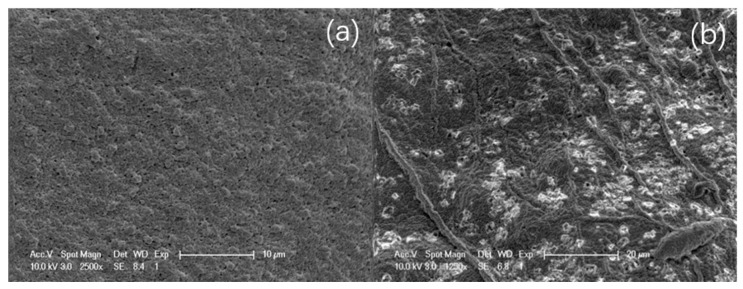
Scanning electron microscopy (SEM) images of the surface of the bio-microcapsules. (**a**) Magnification: 2500×, (**b**) Magnification: 1250×.

According to Figure 2, the microstructure of the microcapsule membrane is homogeneous and dense, the surface morphology is regular with no obvious defects or burrs, the three-dimensional gel grid structure is clear, and the membrane can be seen clearly. Small holes are evenly distributed and oval, with a uniform size; thus, the microcapsules should show good transmission performance, which is conducive to the exchange of material into and out of the membrane.

**Figure 3:**
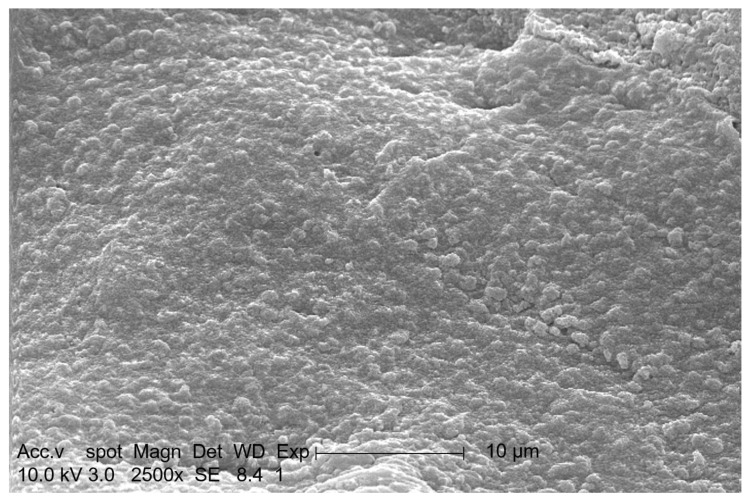
SEM image of the interior of the bio-microcapsules.

Figure 3 shows the SEM images of the microcapsules. It can be seen that the embedded yeast has an oval shape, and the microcapsules are evenly and densely distributed. When the microcapsules are in strong acidic, strong alkaline, or toxic and harmful pollutant environments, the acid, alkali and toxic pollutants form a concentration gradient from the capsule surface due to internal diffusion [33,34]; therefore, the high internal density of the bacteria is conducive to improving their anti-toxic, anti-acid and anti-alkali capacity.

#### 3.2.2. FTIR

**Figure 4:**
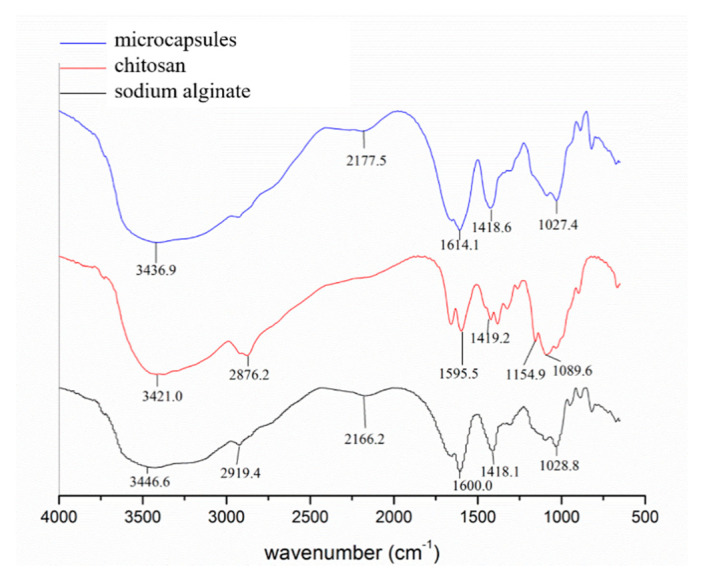
Infrared spectroscopy.

The chemical structures of the microcapsules were confirmed by FTIR (Figure 4). The C–H absorption peak of the six-membered ring of the sodium alginate macromolecule was observed at 2919.4 cm^−1^. The complex structure limits the stretching vibration of C–H on the six-membered ring, so the change of dipole moment is small and the absorption peak is weakened.

For the FTIR spectrum of sodium alginate, the characteristic peaks at 1600 cm^−1^ and 1418.1 cm^−1^ are assigned to the stretching vibration of the –COO– group [22]. The peak at 1028.8 cm^−1^ contributes to the C–OH stretching vibration. For chitosan, the characteristic band is the acylamino in the chitosan matrix, which shows two absorbance peaks—1649 cm^−1^ (C–O) and 1595 cm^−1^ (N–H), which are attributed to the characteristic absorption of amides I and II, respectively [35].

As to the spectrum of the microcapsules, the peak at 1614.1 cm^−1^ revealed that the –COO– group within the sodium alginate overlaps with the N–H group in chitosan at 1595.5 cm^−1^. Similarly, the electrostatic interaction between the groups of C–OH and NH_3_^+^ on the surface of the prepared microcapsules leads to the disappearance of a small peak at 1027.4 cm^−1^, indicating that the prepared product is a chitosan sodium alginate microcapsule. The difference between the microcapsule and the sodium alginate infrared spectrum shows that the alginate macromolecule reacted with the calcium ion after dripping the sodium alginate into the CaCl_2_ solution, and the Ca^2+^ complexed with the sodium alginate to form the coordination structure.

It can be seen from Figure 4 that the hydroxyl vibration peaks of the pure chitosan and the pure sodium alginate were 3421.0 cm^−1^ and 3446.6 cm^−1^, respectively, while the peak of the hydroxyl stretching vibration of the microcapsule was 3436.9 cm^−1^, shifting in the direction of low wavenumbers with the increasing of peak intensities. The interaction between chitosan and sodium alginate was enhanced, and positive and negative ions were formed by hydrogen bonding.

At the same time, the O–H stretching vibration peak in the microcapsules broadened, which indicates that only some of the hydroxyl groups were involved in coordination. The other hydroxyl groups were associated with each other, forming absorptions at higher wavenumbers and overlapping peaks at low wavenumbers. This is apparently due to the formation of chitosan molecules between the hydrogen bond polymerization and the formation of glutaraldehyde cross-linked chitosan.

### 3.3. Removal of Turbidity

**Figure 5:**
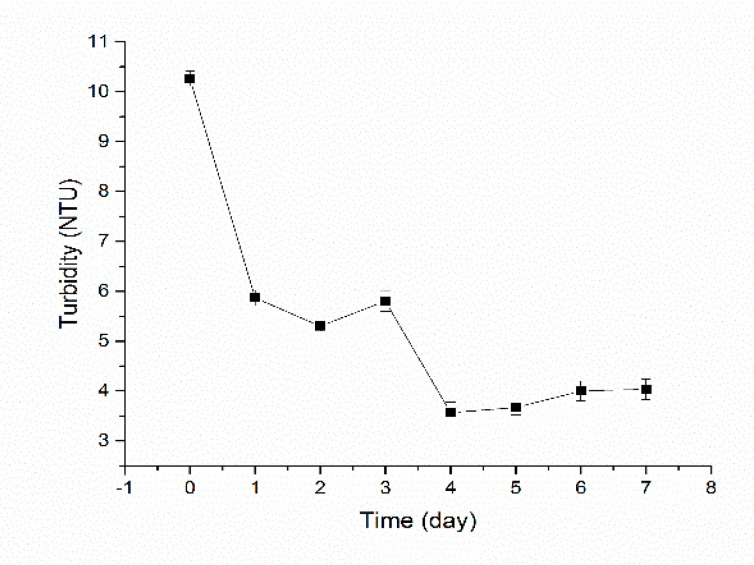
Removal of turbidity.

It can be seen from Figure 5 that the biological microcapsules removed 43% turbidity after one day when treated with raw water. Between four and seven days, the removal rate reached 61–65%. At the end of the process, some yellow-brown floc deposits were found in the contact reaction zone, indicating that bio-microcapsules promoted flocculation and reduced the turbidity.

In drinking water treatment, the main material of the microcapsule, namely, sodium alginate, is an excellent coagulant [36]. The linear polymer structure can move through the water film and can generate an adsorption bridge effect between particles by the functional groups within. Chitosan is a U.S. EPA(Environmental Protection Agency)-approved drinking water purification agent, which is considered one of the organic flocculants with the most potential [37]. The mechanism of flocculation is a combination of electrical neutralization and adsorption bridging. In a diluted acid solution, the amino group on the chitosan molecular chain is protonated, which results in the surface of the chitosan becoming positively charged, neutralizing the negative charge on the colloid surface and reducing its zeta potential, thus destabilizing the colloid [36,38]. As an organic macromolecule, chitosan also affects the adsorption bridge effect in flocculation. It is generally believed that adsorption bridging plays a key role in flocculation; the efficiency of adsorption bridging is determined by the viscosity of chitosan, whereas relative molecular mass influences viscosity [39]. Therefore, the larger the molecular weight, the stronger the adsorption bridge effect; thus, the flocculation effect is enhanced. As a result, when the biological microcapsules are added to the water, the suspended particles may flocculate due to the characteristics of the material.

### 3.4. Removal of Nitrite Nitrogen, Nitrate Nitrogen, Ammonia Nitrogen and Total Nitrogen

**Figure 6:**
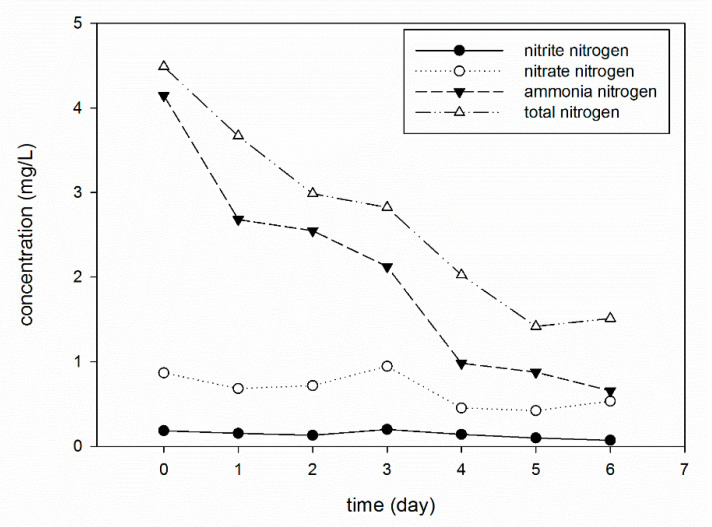
Removal of nitrogen.

To study the removal and transformation of nitrogen, the experimental raw water with an ammonia concentration of 5 mg/L was arranged on the basis of landscape lake water. Total nitrogen in the raw water is the sum of nitrite nitrogen, nitrate nitrogen, ammonia nitrogen, other organic nitrogen, protein nitrogen and other inorganic nitrogen. Ammonia nitrogen in the raw water is predominantly represented by inorganic nitrogen. Figure 6 shows the removal rates of nitrite nitrogen, nitrate nitrogen, ammonia nitrogen and total nitrogen during the experiment.

As a small inorganic molecule, the removal of ammonia nitrogen mainly relies on biological treatment. There are two main biodegradation pathways: one is the direct synthesis of essential nutrients involving ammonia nitrogen, such as nucleic acids, proteins, and other nitrogen-containing organic matter; the other is through the nitrification of ammonia nitrogen into NO_3_^−^ and NO_2_^−^, and the further degradation of N_2_ through denitrification [40].

This process is mainly dependent on nitrification, nitrification bacteria and the denitrifying bacteria metabolism. In addition, there are also bacteria, such as *Arthrobacter* and *Bacteroides sp.*, that can provide ammonia nitrogen oxidation for NO_2_^−^ and NO_3_^−^, but they do not rely on ammonia to obtain energy, as nitrifying bacteria do. In conventional biological pretreatment, the second nitrification and the denitrification pathway are more common, but despite the beneficial effect of ammonia removal, it may also induce the accumulation of nitrate and nitrite nitrogen defects.

The concentration of NH_3_–N decreased continuously in both of the contact reaction zones, while the concentration of nitrite decreased slightly, but almost always coincided with the initial concentration. During the mid-run period, there was some accumulation, which later decreased again, and the overall removal rate fluctuated between 10% and 30%. The degradation of ammonia nitrogen by yeast operates via the first biodegradation pathway mentioned above, namely, via assimilation (Equation (1)). The heterotrophic bacteria assimilate ammonia nitrogen into the organism. The process is different from that of nitrification and nitrifying bacteria, which regards ammonia nitrogen as an energy donor, followed by the oxidation of NO_3_^−^ and NO_2_^−^ for chemical energy autotrophic metabolism (Equations (2) and (3)), so that the nitrite nitrogen and nitrate nitrogen concentrations do not change significantly.

(1) $$\begin{array}{c} nC_{x}H_{y}O_{z}+nNH_{3}+n(x+\frac{y}{4}-\frac{z}{2}-5)O_{2}\to \\ {\left(C_{5}H_{7}NO_{2}\right)}_{n}+n\left(x-5\right)CO_{2}+\frac{n}{2}\left(y-4\right)H_{2}O \end{array}$$

(2) $$2NH_{4}^{+}+O_{2}\to 2NO_{2}^{-}+4H^{+}+2H_{2}O+\mathrm{energy}$$

(3) $$2NO_{2}^{-}+O_{2}\to 2NO_{3}^{-}+\mathrm{energy}$$

Some studies have shown that *Candida* has a certain effect on the removal of nitrite from aquaculture water [41,42], but it requires a rich organic nutrient substrate in water. Therefore, the small fluctuation in the concentrations of NO_2_–N and NO_3_–N in the water is caused by microbial nitrification and denitrification. This indicates that the yeast is competitive in the water body, and the degradation of NH_3_–N is still dominated by assimilation. Furthermore, the sum of the inorganic nitrogen was clearly lower than that of the TN concentration in the effluent, indicating the presence of organic nitrogen. The biodegradation of ammonia nitrogen produces a lot of protein and amino acid organic nitrogen as its own nutrient.

### 3.5. Effect of Water Quality Fluctuations on Removal Efficiency

#### 3.5.1. Effect of a High Concentration of NH_3_–N

**Figure 7:**
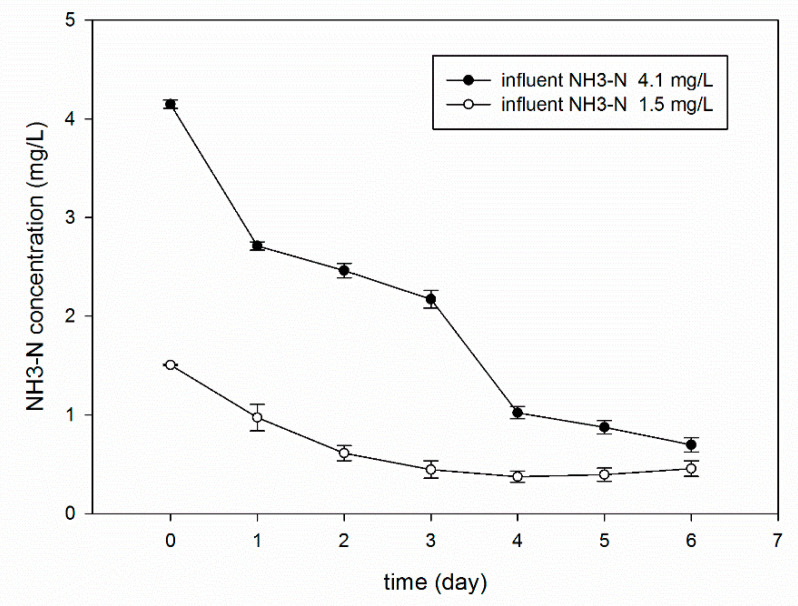
Removal of NH_3_–N at different influent concentrations.

In a real waterbody, the NH_3_–N concentration in raw water displays seasonal fluctuations. A high concentration of NH_3_–N occurs in winter, and ammonia concentration is lower in summer due to active microorganisms. As known, ammonia is an essential nitrogen source for microorganisms, but it is considered an inhibitor at high concentrations [43]. The inhibition of anaerobic bacteria under high ammonia concentrations has often been reported [44]. Therefore, it is necessary to investigate whether the bio-microcapsules can withstand the impact load of pollutants, and can deal with unexpected pollution events. Figure 6 shows the removal of NH_3_–N with the same dosage of microcapsules at two influent ammonia nitrogen concentrations (1.5 and 4.1 mg/L). From Figure 7, it can be seen that the degradation rate is faster when the concentration of NH_3_–N fluctuates in the range of 1–5 mg/L. When the concentration of NH_3_–N was approximately 1.5 mg/L, the removal rate was 36% after one day, reaching 75% after four days. When the concentration of NH_3_–N exceeded four mg/L, the removal rate was 34% after one day, while reaching 83% after six days. The results suggest that both of the tested ammonium concentrations supported the growth of CICC1351. The activity of the most nitrifying bacteria has been reported to be inhibited by high concentrations of ammonium [45,46]. The growth of microorganisms could be inhibited both by total ammonia and by free NH_3_ [44]. Thus, the yeast strain CICC1351 has potential applicability in microcapsules due to its high ammonium tolerance.

#### 3.5.2. Effect of a High Concentration of Organic Matter

**Figure 8:**
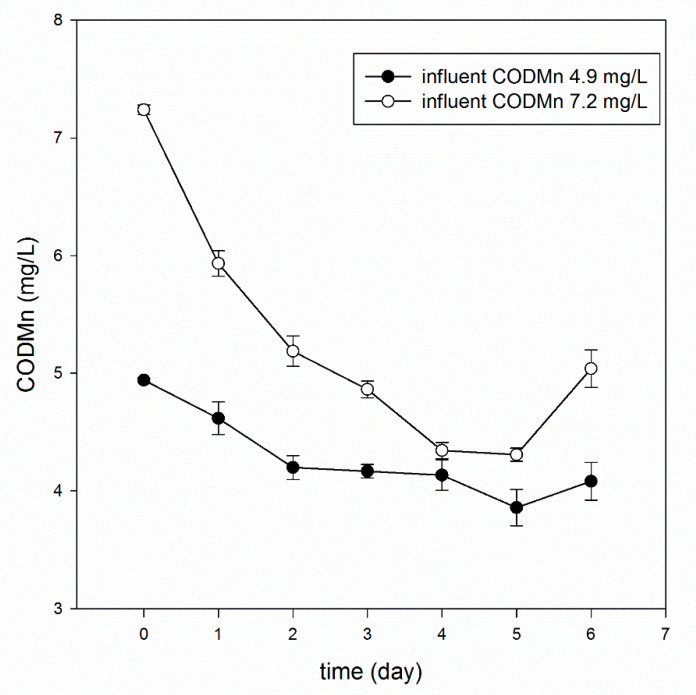
Removal of COD_Mn_ under different influent concentrations.

The removal rate of COD_Mn_ varied with the influent concentration of COD_Mn_, as shown in Figure 8. The COD_Mn_ removal rate reached 40% when the influent concentration was high (7.2 mg/L), and 22% when the influent concentration was low (4.9 mg/L). The conclusion is inconsistent with Yang [44] et al. (a stable COD removal over 80% was acquired over a period of nearly 2 months, in spite of variation of the influent COD). This result shows that when the biological respiration of yeast on the water nutrient substrate is relatively high, there is more organic matter in the water, it can provide more nutrients, and the degradation effect is improved. Yeast strains of differing origins have different pollutant removal abilities [47], and isolated yeast strains obtained by spontaneous selection pressure in wastewater often better reduce COD [11,48]. Because of the special membrane structure of the microcapsules, only small molecules can enter their interior [49], make contact with the yeast, and start to degrade, which also limits the removal of organic matter. In general, the bio-microcapsules were more stable in removing organic compounds from micro-polluted raw water.

#### 3.5.3. Effect of Temperature

Temperature significantly affects the microbial community [50]. Temperature also plays an important role in the growth of microorganisms, and can influence their metabolism by affecting the enzyme activity in microorganisms. Traditional biological pretreatment is subject to the impact of temperature, which is a significant drawback of this method. A good processing effect can only be obtained in a suitable temperature environment, as cell enzyme activity is high and metabolism is strong. In practical engineering, the removal efficiency of ammonia nitrogen in traditional biological treatment during winter is greatly reduced. To investigate whether the biological tolerance of the microcapsule is changed in the special embedding environment, and consequently whether it can overcome the shortcomings of traditional biological treatment, the water removal efficiency of ammonia water was measured.

**Figure 9:**
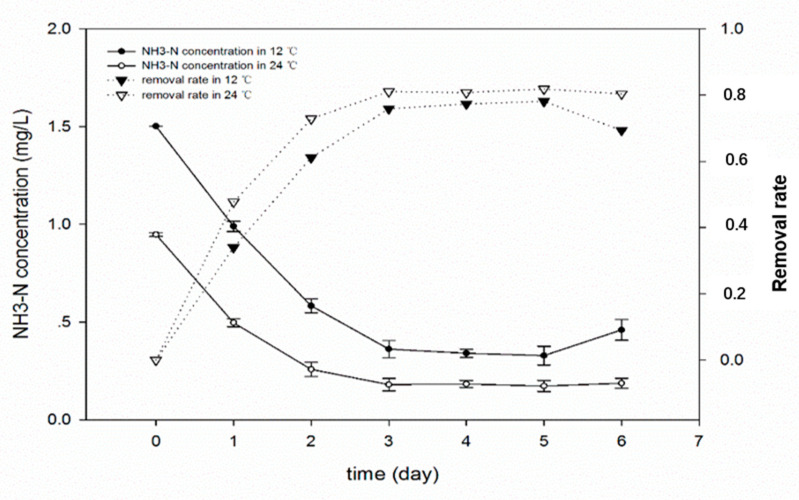
Removal of NH_3_–N at different temperatures.

Condition 1 refers to the raw water of Sanhaowu at 12 °C in early spring, while Condition 2 to the raw water of Sanhaowu at 24 °C in early summer. Figure 9 shows that when the yeast cells are embedded in the bio-microcapsules, the temperature is lower than the optimal growth temperature of 28 °C, and the temperature difference between the two is doubled. As to the removal rate of ammonia nitrogen, the difference is not big, both can exceed 75%, but the lower the temperature conditions are, the slightly slower will be the initial degradation efficiency of ammonia nitrogen, and this is also related to the high ammonia nitrogen content in low-temperature water. The optimal temperature for yeast growth is 28–30 °C [51]. At this temperature, the activity of protease is at its maximum, metabolism is strong, and the organic matter can be synthesized by ammonia nitrogen. For bulk cells, if the temperature is too low (usually <15 °C), the enzyme activity is significantly reduced [52]. Clearly, bio-microcapsules provide a specific environment for yeast cells to enhance their ability to interfere with the external environment, even at low temperatures, and the degradation of ammonia nitrogen can be maintained at a certain level. Therefore, bio-microcapsule pretreatment technology has certain advantages for resisting changes in external temperature, and the special embedded structure provides a buffer for the bacteria inside the capsule, which can ensure microorganism activity at lower temperatures.

## 4. Conclusions

The raw water treatment experiments showed that yeast-chitosan bio-microcapsules have unique advantages in the treatment of micro-polluted surface water. Unlike nitrifying bacteria, *Candida tropicalis* can rapidly and effectively remove suspended solids and ammonia nitrogen. Moreover, the bio-microcapsule pretreatment process is capable of resisting impact loads and fluctuations in water quality. Even at a low temperature (12 °C), the removal rate of ammonia nitrogen reached 79%. It did not lead to a temporary increase of nitrite concentration, nor to excessive accumulation of nitrogen. The application of bio-microcapsules is simple; it only requires aeration and certain nutrient substrates, and can be adapted to raw drinking water with a poor nutrient substrate. Therefore, *Candida tropicalis* bio-microcapsules have a promising value in future engineering applications.

## Figures and Tables

**Table 1:** Water quality index of Sanhaowu River.

COD_Mn_ (mg/L)	TOC (Total Organic Carbon) (mg/L)	NH_3_-N (mg/L)	TN (Total Nitrogen) (mg/L)	UV_254_ (Ultraviolet 254) (cm^−1^)	pH (-)	Turbidity (NTU)
4.944	4.14	1.504	2.486	0.090	7.10	15.3

**Table 2:** Orthogonal experiments on the four factors affecting the mechanical properties of the bio-microcapsules.

Experiment Number	Factor Level				Mechanical Strength (5 Capsules)/(g·cm^−2^)	Shape Description
	A	B	C	D		
1	1.5	1.0	15	5	23.71	uneven, long tail
2	1.5	2.0	20	10	42.10	as above
3	1.5	4.0	30	15	49.67	as above
4	1.5	5.0	45	20	44.10	as above
5	2.0	1.0	20	15	174.58	basically uniform with a small tail
6	2.0	2.0	15	20	206.17	as above
7	2.0	4.0	45	5	361.73	as above
8	2.0	5.0	30	10	585.38	as above
9	2.5	1.0	30	20	94.88	as above
10	2.5	2.0	45	15	264.53	basically uniform, some with a small tip
11	2.5	4.0	15	10	156.51	as above
12	2.5	5.0	20	5	304.88	as above
13	3.0	1.0	45	10	252.64	spherically uniform
14	3.0	2.0	30	5	288.13	as above
15	3.0	4.0	20	20	555.22	as above
16	3.0	5.0	15	15	307.95	as above
K1	39.90	136.45	173.59	244.61	-	
K2	331.97	200.23	202.42	259.16		
K3	205.20	280.78	254.52	199.18		
K4	350.99	310.58	230.75	225.09		
k1	9.97	34.11	43.40	61.15		
k2	82.99	50.06	50.61	64.79		
k3	51.30	70.20	63.63	49.80		
k4	87.75	77.64	57.69	56.27		
R	77.77	43.53	20.23	14.99		

Notes: A = concentration of sodium alginate solution (%); B = calcium chloride solution concentration (%); C = solidifying time (min); D = coating time (min).
